# GSK-3β, FYN, and DYRK1A: Master Regulators in Neurodegenerative Pathways

**DOI:** 10.3390/ijms22169098

**Published:** 2021-08-23

**Authors:** Stefania Demuro, Rita M. C. Di Martino, Jose A. Ortega, Andrea Cavalli

**Affiliations:** 1 Computational and Chemical Biology, Italian Institute of Technology, 16163 Genoa, Italy; stefania.demuro@iit.it (S.D.); rita.dimartino@iit.it (R.M.C.D.M.); jose.ortega@iit.it (J.A.O.); 2 Department of Pharmacy and Biotechnology, University of Bologna, 40126 Bologna, Italy

**Keywords:** protein kinases (PKs), central nervous system (CNS), blood-brain barrier (BBB), tauopathies, PKs modulation, crosstalk, multi-target

## Abstract

Protein kinases (PKs) have been recognized as central nervous system (CNS)-disease-relevant targets due to their master regulatory role in different signal transduction cascades in the neuroscience space. Among them, GSK-3β, FYN, and DYRK1A play a crucial role in the neurodegeneration context, and the deregulation of all three PKs has been linked to different CNS disorders with unmet medical needs, including Alzheimer’s disease (AD), Parkinson’s disease (PD), frontotemporal lobar degeneration (FTLD), and several neuromuscular disorders. The multifactorial nature of these diseases, along with the failure of many advanced CNS clinical trials, and the lengthy approval process of a novel CNS drug have strongly limited the CNS drug discovery. However, in the near-decade from 2010 to 2020, several computer-assisted drug design strategies have been combined with synthetic efforts to develop potent and selective GSK-3β, FYN, and DYRK1A inhibitors as disease-modifying agents. In this review, we described both structural and functional aspects of GSK-3β, FYN, and DYRK1A and their involvement and crosstalk in different CNS pathological signaling pathways. Moreover, we outlined attractive medicinal chemistry approaches including multi-target drug design strategies applied to overcome some limitations of known PKs inhibitors and discover improved modulators with suitable blood–brain barrier (BBB) permeability and drug-like properties.

## 1. The Neurokinome in Drug Discovery

The human kinome plays a crucial role in many physiological events, and its dysregulation is associated with a large portion of multifactorial disorders, including cancer and neurodegenerative diseases. Among these latter ones, neurodegeneration has a dramatic impact on the aging population. Protein kinases (PKs) represent very attractive and challenging drug targets for industry and academia to tackle complex disorders affecting peripheral and central tissues [1,2]. However, the development of PK-targeted therapies in neuroscience has not been primarily investigated due to several issues, including the multifactorial nature of the central nervous system (CNS) diseases, the failure of many advanced CNS clinical trials, and the lengthy approval process of a novel CNS drug by the U.S. Food and Drug Administration (FDA) [3,4].

CNS drug discovery places unique challenges linked to the blood–brain barrier (BBB) and the paucity of translational animal models to test new drug candidates [5,6]. The BBB has been extensively investigated as a dynamic and selectively protective membrane responding to changes in its environment and as part of a more complex neurovascular unit in which endothelial cells, astrocytes, pericytes, and neurons interact to restrict the flow of native and foreign agents between the blood and the CNS. BBB reflects the properties of two components: one forms a structural/physical barrier composed of endothelial cells and extremely tight intercellular junctions that regulate diffusion of solutes between blood and brain; the other is a biochemical/selective barrier due to the presence of specific transport proteins expressed on the luminal (blood-facing) and abluminal (brain-facing) plasma membranes of the endothelial cells able to act as important CNS gatekeepers, selectively increasing brain permeability to essential nutrients or effectively preventing foreign compounds’ permeation [5]. A key element of the BBB is P-glycoprotein (P-gp), which is an ATP-driven efflux pump localized to the luminal (blood side) plasma membrane, which handles a vast range of substrates, in the range of 300 to 4000 Da in mass. It restricts the penetration of drugs of the brain, greatly influencing CNS pharmacotherapy, and contributes to cross-resistance to commonly prescribed drugs, including multiple classes of chemotherapeutics [7,8].

CNS drugs must penetrate the BBB, reaching a brain concentration adequate for target engagement and modulation. Therefore, particular attention must be devoted to the factors that hinder the passive BBB penetration of a molecule in the CNS drug discovery, including large size, high topological polar surface area, and a high degree of hydrogen bonding. Size and lipophilicity are two crucial properties influencing the brain exposure and efficacy of a CNS drug candidate. Often, a balance must be found between decreasing size and increasing lipophilicity to make a drug more penetrating while simultaneously avoiding both efflux mechanisms (e.g., P-gp-mediated efflux) and drug sequestration elsewhere in the body (e.g., plasma proteins, fatty tissue) [6].

Ghose et al., analyzing the physicochemical property and the chemical structural profiles of several CNS and non-CNS oral drugs in a comparative fashion, provided guidelines for designing high-quality CNS drugs. According to their property distribution study and the classification tree, a compound with an ideal property profile should possess a topological molecular polar surface area of <76 Å^2^ (25–60 Å^2^), at least one nitrogen (including one aliphatic amine), fewer than seven (two to four) linear chains outside of rings, less than three (preferred zero or one) polar hydrogen atoms, the volume of 740–970 Å^3^, the solvent accessible surface area of 460–580 Å^2^, and a positive QikProp (QP) CNS parameter (https://www.schrodinger.com/products/qikprop, accessed on 26 July 2021) [9]. An additional approach toward assessment of drug-likeness properties affecting overall brain permeability and very useful in prioritizing lead candidates consists in the Pfizer’s CNS multiparameter optimization [10], which takes into account calculated partition coefficient (ClogP), calculated distribution coefficient at pH 7.4 (ClogD), molecular weight (*M*_W_), acid dissociation constant (pK_a_) of ionizable groups, together with total polar surface area and the number of hydrogen bond donors. 

The safety and efficacy of a CNS drug candidate must be tested in an animal model that displays relevant disease characteristics before proceeding to human clinical trials. However, the development of validated animal models for CNS-related disorders is hampered by their complex and least understood etiologies and the difficulty of reproducing in the same model all the disease hallmarks. In the past 20 years, several efforts have been devoted to developing animal models for Alzheimer’s disease (AD), and extensive research has been focused on “curing” animals genetically predisposed to generate amyloid β (Aβ) plaques or neurofibrillary tangles (NFTs) of τ protein. Unfortunately, no results have been translated from models into human clinical trials so far [6]. 

**Figure 1:**
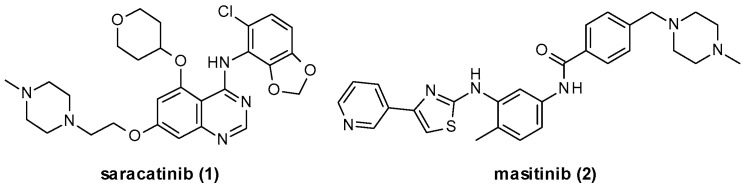
Chemical structures of saracatinib (1) and masitinib (2), two FYN inhibitors firstly developed for the treatment of cancer and repurposed as CNS-agents.

A cost-effective and reduced-risk strategy widely exploited in the last decade to overcome the trickiness of CNS drug discovery is drug repurposing. The rediscovery of “old molecules” fits with the need for poorly addressed therapeutic areas in which the CNS-related pathologies represent a leading field [11,12]. Great examples in this scenario are saracatinib (1) and masitinib (2), which are two well-known inhibitors of FYN kinase firstly developed as anti-cancer agents. Compound 1 (Figure 1), also known as AZD0530, is a highly selective Src family kinases (SFKs) inhibitor developed by AstraZeneca in 2006 [13]. Unfortunately, it failed in phase II clinical trials because of its limited therapeutic benefits. The excellent pharmacokinetic properties and high BBB permeability of saracatinib encouraged its repurposing as a promising CNS agent for AD treatment [14]. Likewise, compound 2 (Figure 1) is a potent and selective tyrosine kinase inhibitor targeting mainly wild-type and mutated c-Kit receptor (c-KitR). It is the first anti-cancer therapy approved in veterinary medicine for the treatment of unresectable canine mast cell tumors (CMCTs) and currently is under study for the treatment of mild to moderate AD [15,16]. Both cases will be discussed more deeply in Section 10 on inhibition of FYN kinase.

The multifactorial nature of CNS-related diseases requires innovative strategies, which may help to overcome some limits of single-target small-molecule ligands. Against this scenario, multi-target compounds are emerging as promising approaches to CNS-related pathologies, bearing other levels of complexity, such as balancing the multi-target profile [17]. Moreover, this approach can reduce the possibility of developing drug resistance. Several multi-target agents have been rationally designed by applying a multi-target drug design approach. Others, such as single kinase inhibitors, were subsequently found to be multi-target inhibitors because of the structural homology among the ATP-binding site of other kinases [18].

## 2. GSK-3β, FYN, and DYRK1A, Emerging Targets in the Neurokinome

GSK-3β, FYN, and DYRK1A represent three closely related PKs widely investigated within the neurokinome context due to their pivotal roles in both the onset and development of complex CNS-related diseases, including neurodegenerative (e.g., AD, Pick’s disease (PiD), frontotemporal lobar degeneration (FTLD), Parkinson’s disease (PD), amyotrophic lateral sclerosis (ALS)) and neuromuscular disorders (e.g., spinal muscular atrophy (SMA) and myotonic dystrophy type 1 (DM1)) [19,20,21,22,23,24,25,26]. Despite the different clinical manifestations, common pathogenic mechanisms, including oxidative stress, abnormal protein deposition, mitochondrial deficit, glutamate excitotoxicity, and neuroinflammation have been observed, pointing to converging pathways in neurodegeneration [27,28]. In this multifaceted pathological scenario, the deregulation of all three kinases has been recognized as a key event. Regulatory crosstalk by these PKs on different pathological signaling pathways has been elucidated, suggesting the great potential of the simultaneous modulation of different nodes of the neurodegeneration network to achieve disease-modifying effects.

### 2.1. GSK-3β

**Figure 2:**
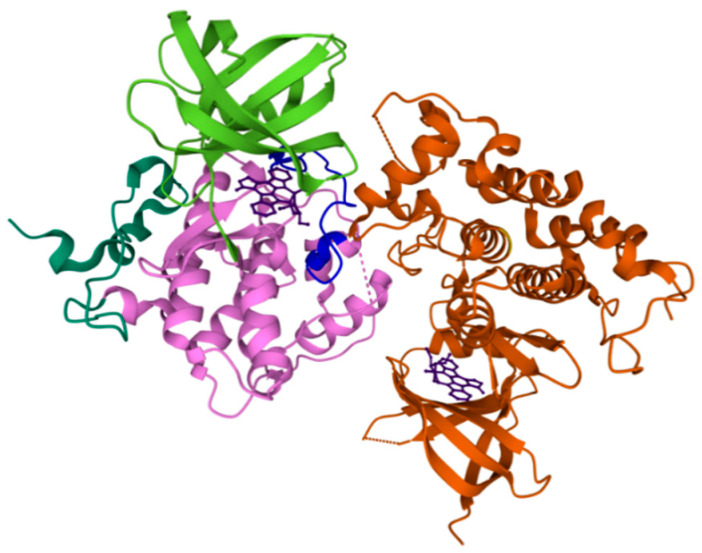
Stereo pair secondary structure cartoon of human GSK-3β in complex with staurosporine (**3**) (purple) PDB ID: 1Q3D [31]. The β-strand domain at the *N*-terminal end is depicted in light green, the α-helical domain at the *C*-terminal end is depicted in pink, and the activation loop is depicted in blue.

Glycogen synthase kinase-3 (GSK-3, Figure 2) is a multitasking Ser/Thr kinase primarily expressed in CNS and involved in regulating several cellular processes, including cellular division, proliferation, differentiation, and adhesion. It is intimately implicated in the control of apoptosis, synaptic plasticity, axon formation, and neurogenesis in neurons. This enzyme phosphorylates more than a hundred different substrates, and several homologs have been identified in different organisms such as fungi, microorganisms, etc. [19,29].

In 1980, it was isolated from rabbit skeletal muscle and recognized as one of the five enzymes involved in glycogen synthase phosphorylation. In mammalian cells, this PK exists in two different isoforms, namely GSK-3α (51 kDa) and GSK-3β (47 kDa), which are ubiquitously expressed in the brain, with high levels of expression in the hippocampus, cerebral cortex, and the Purkinje cells of the cerebellum, even if the expression ratio of these two isoforms favors GSK-3β. The first crystal structures of the last-mentioned isoform were published in 2001 and have assisted in showing this enzyme made by a typical two-domain kinase fold composed of a β-strand domain (residues 25–138) and a α-helical domain (residues 139–343) at the *N*- and *C*-terminal ends, respectively. The ATP-binding site is positioned at the interface of the α-helical and β-strand domains and is bordered by the glycine-rich loop and the hinge. The activation loop (residues 200–226) runs along the surface of the substrate binding groove, and the β-strand domain includes a short helix (residue 96–102), which is highly conserved in all kinases and encompasses two residues, Arg96 and Glu97, which are mainly involved in the catalytic activity of the protein. Moreover, at the entrance of the GSK-3β ATP binding site, Cys199 has been recognized to play a key role in the irreversible or pseudo-irreversible inactivation of the enzyme by covalent interaction (via sulfur–carbon bond formation) [30].

**Figure 3:**
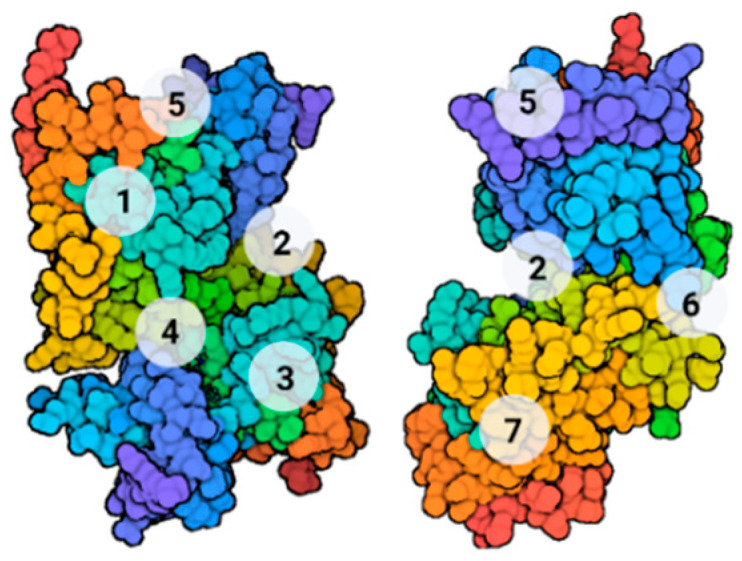
Representation of the seven well-conserved cavities of GSK-3β (PDB ID: 1Q3D) characterized from Palomo et al. [32] (created with BioRender.com).

In 2011, Palomo et al., searching for new druggable sites on the enzyme, identified seven well-conserved cavities on the surface of 25 PDB different structures of GSK-3β by employing the free geometry-based algorithm fpocket and hpocket programs. Three of these pockets correspond to the known binding sites of the enzyme: ATP (1), substrate (2), and peptides axin/fratide (3), while the other four are new cavities situated on the *N*-terminal lobe of the kinase (5), in the hinge region between the *C-* and *N*-terminal lobes (6), and finally two on the *C*-lobe of the enzyme (4 and 7, Figure 3) [32].

In general, the phosphorylation of specific amino acid residues such as Tyr216 within the activation loop of GSK-3β induces a conformational change and consequent increase of the enzyme activity. However, this PK can also achieve a catalytically active conformation without a specific phosphorylation [33,34]. Concerning the functional aspects, GSK-3β requires previous phosphorylation of its substrates by priming kinases and, unlike other PKs, is constitutively active in resting conditions and is inhibited in response to upstream signals [35]. Post-translational phosphorylation at Ser9 is associated to enzyme inhibition, while dephosphorylation at specific inhibitory sites by different phosphatases such as protein phosphatase 1 (PP1) leads to PK activation [33,36].

Abnormal regulation of GSK-3β has been linked to both the onset and progression of different chronic conditions such as diabetes, cancer, neurodegenerative (e.g., AD, PD, ALS), and behavioral diseases (e.g., bipolar disorder (BD), major depression (MD), schizophrenia). In CNS-related disorders, aberrant GSK-3β activity is associated with the dysregulation of different proteins, namely microtubule-associated protein τ, presenilins, amyloid precursor protein (APP), collapsin response mediator proteins, components of the Wnt signaling pathway, β-catenin, and heat shock proteins [37]. Moreover, several lines of evidence have reported GSK-3β as a mediator of neuroinflammatory processes. These contribute to the progressive impairment of cognitive and/or motor functions associated with neurodegenerative diseases such as AD, PD, and Huntington’s disease (HD) [36,38].

### 2.2. FYN

FYN is a non-receptor Tyr kinase (TK) identified and characterized in 1988 [39]. It belongs to the SFKs as part of the subfamily SrcA, together with Yes and Src enzymes. It is a 59 kDa protein consisting of 537 amino acids and is encoded by a gene located on chromosome 6q21. FYN mediates various cellular processes, including the T-cell receptor signaling pathway, regulation of brain function, adhesion-mediated signaling, and cell survival [40]. It prevails in many brain areas and is involved in both development and adult brain physiology. Moreover, the same PK plays a unique role in CNS myelination by coupling with cell surface proteins, including myelin-associated glycoprotein. Consistent with these functions, FYN knockout mice have significantly reduced brain myelination, disrupting hippocampal architecture, impaired spatial learning, and increased sensitivity to ethanol [41].

**Figure 4:**
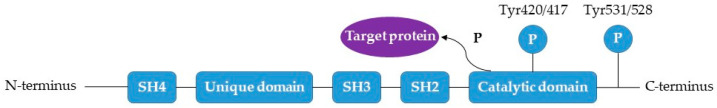
Schematic representation of FYN isoforms FYN-B and FYN-T.

Three different FYN isoforms have been identified: FYN-B is mainly expressed in the CNS, FYN-T is mainly expressed in hematopoietic cells (T-cells), and FYN-Delta7 is mainly expressed in peripheral blood mononuclear cells [42]. As all SFK members, the FYN structure is characterized by six different domains [24,43,44]: the Src homology (SH) domains SH1 (catalytic domain), SH2, SH3, SH4, the so-called unique domain, and a *C*-terminal regulatory region (Figure 4). All FYN isoforms share the catalytic domain SH1; however, FYN-B and FYN-T differ in the linker sequence between SH1 and SH2, while FYN-Delta7 presents a deletion of residues 233–287 when compared to FYN-B [42,45]. SH3 interacts with proline-rich sequences on target substrates and is involved in the autoinhibition regulatory mechanism. At the same time, the unique domain is specific for each SFK member and is responsible for particular proteins interactions. 

FYN activity is regulated through interdomain interactions, which in turn is influenced by Tyr residues phosphorylation and dephosphorylation processes. The phosphorylation of a specific *C*-terminal domain Tyr residue (Tyr531 for FYN-B and Tyr528 for FYN-T) induces a global close protein conformation due to the SH2 domain engagement, leading to the non-accessibility of the catalytic domain. On the other hand, the phosphorylation of a distinct Tyr residue in the catalytic domain (Tyr420 for FYN-B and Tyr417 for FYN-T) improves the enzyme activity (Figure 4). 

FYN overexpression has been widely correlated with cancer onset due to the enzyme pivotal role in the morphogenetic transformation and cell growth; nevertheless, recent studies and preclinical evidence have reported the same protein kinase involvement in different neurodegenerative disorders, including AD and PD [43,46].

### 2.3. DYRK1A

**Figure 5:**
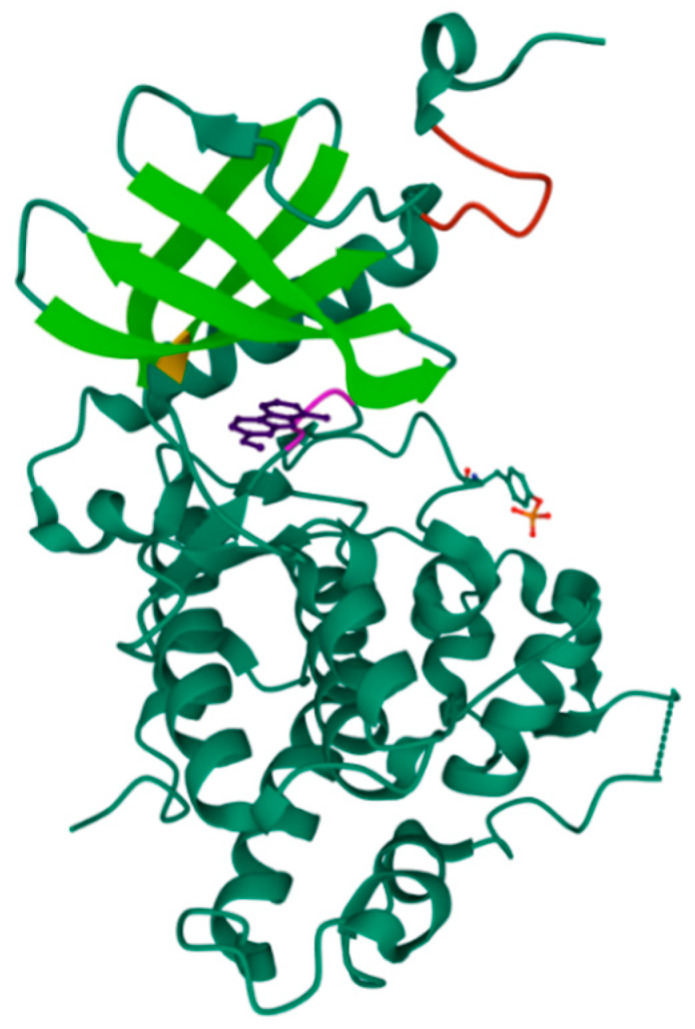
Stereo pair secondary structure cartoon of human DYRK1A in complex with harmine (4) (purple) PDB ID: 3ANR [51]. Five antiparallel β-strands at the *N*-terminal lobe are depicted in light green; the DH box is depicted in red; Phe238 is depicted in orange; the DFG pocket is depicted in magenta.

Dual-specificity tyrosine phosphorylation-regulated kinase 1A (DYRK1A, Figure 5) belongs to a family of dual-specificity protein kinases (DYRK kinases) that possess Ser and Thr phosphorylation activity as well as autophosphorylation activity on Tyr residues [25,47]. DYRKs family is part of the CMGC group of Ser/Thr kinases, which also includes cyclin-dependent kinases (CDKs), mitogen-activated protein kinases (MAPKs), glycogen synthase kinases (GSKs), and CDC2-like kinases (CLKs). DYRK members participate in critical signaling pathways that control postembryonic neurogenesis, developmental processes, cell survival, differentiation, and death [48].

DYRK1A plays a key role in the neural proliferation and neurogenesis of the developing brain, and its gene is located on chromosome 21 (21q22.2), which is a region known as the Down syndrome critical region (DSCR). Due to its location, triplication of the DYRK1A locus in Down syndrome (DS) results in a 1.5-fold increase of DYRK1A mRNA and protein levels in the fetal and adult brain. Under-/over-expression in mammals of DYRK1A gene or mutations in the orthologous gene minibrain (mnb) of Drosophila have been associated with severe retardation of CNS development and maturation [48,49]. Moreover, the upregulation of DYRK1A has been reported to contribute to altered neuronal proliferation in DS through the specific phosphorylation of p53 at Ser15 [50].

As all DYRKs, the DYRK1A isoenzyme contains a conserved catalytic kinase domain, which is centrally located in its primary structure, preceded by the *N*-terminal motif DYRK-characteristic known as DYRK homology (DH) box. It rapidly autoactivates during folding by phosphorylation on Tyr321, the second Tyr residue of the conserved activation loop YxY motif [48]. The kinase domain comprises an *N*-terminal lobe (*N*-lobe) with five antiparallel β-strands and a conserved regulatory α C-helix and a larger *C*-terminal (*C*-lobe) consisting of α-helices. The *N* and *C*-lobes are connected by the hinge region [25]. Interestingly, DYRK1A possesses Phe238 as a gatekeeper residue at the narrow channel, bridging the ATP binding pocket and the DFG (aspartate-phenylalanine-glycine)-pocket [52] significant for its allosteric modulation [53].

DYRK1A has attracted interest in cancer therapy given its crucial role in several pathways, including cell proliferation, apoptosis, malignant cells survival [54], and the regulation of cell cycling and differentiation [55]. Starting from the last decade, different human neurodegenerative pathologies and impaired neurogenesis have also been associated with DYRK1A dysregulation [56,57].

## 3. τ Hyperphosphorylation

Abnormal phosphorylation of microtubule-associated protein τ at different sites, including Ser/Thr residues in Ser/Thr-Pro sequences, is one of the major pathological events in AD and other related neurodegenerative diseases, such as FTLD and additional tauopathies [58,59]. The abnormal phosphorylation of τ is the key driver of neurofibrillary degeneration in AD. In vitro kinetic studies of the binding between hyperphosphorylated and normal τ suggested Ser202/396 and Thr205 among the critical phosphorylation sites, which lead to the sequestration of hyperphosphorylated τ into microtubule-associated proteins and its self-aggregation into NFTs [60]. These aggregates trigger a cascade of biological processes, τ cascade, among others, ultimately culminating in neuronal cell death, brain atrophy, and cognitive decline [61].

**Figure 6:**
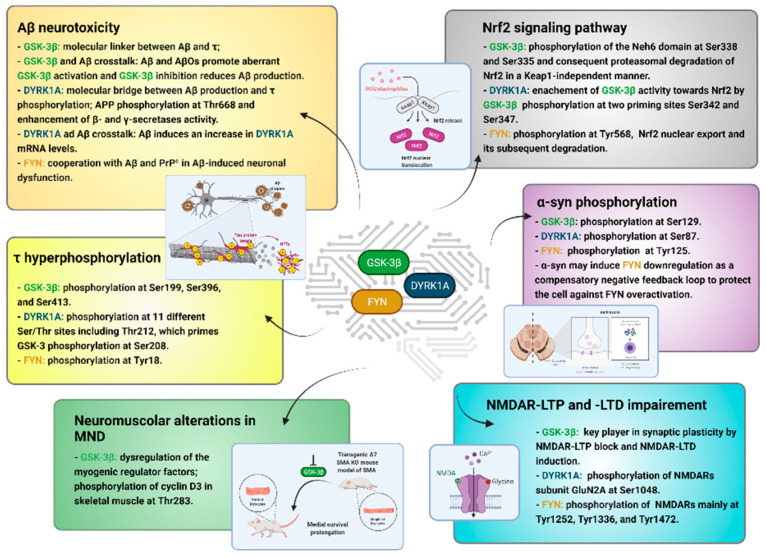
Schematic representation of the general involvement of GSK-3β, FYN, and DYRK1A in neurodegenerative diseases and motor neuron disease (MND) (created with BioRender.com).

In this context, while GSK-3β induces human τ phosphorylation mainly at Ser199, Ser396, and Ser413 [62], DYRK1A phosphorylates 11 different Ser/Thr sites of τ, including Thr212 as the predominant one [26], and FYN, physically linked to the amino-terminal projection domain of τ, is responsible for its phosphorylation at Tyr18 (Figure 6) [63].

Phosphorylation at Thr212 primes τ for phosphorylation by GSK-3β at Ser208 in vitro, suggesting a more general role for DYRK1A in priming phosphorylation of GSK-3β substrates [32]. Additionally, DYRK1A, by phosphorylation of the alternative splicing factor (ASF) at Ser227, Ser234, and Ser238, causes dysregulation of alternative splicing of τ, leading to NFTs formation [64]. Remarkably, τ overexpression has been reported to promote GSK-3β activation and mediate GSK-3β toxicity whereas, in τ absence, the neurodegenerative and cognitive phenotype observed in GSK-3β overexpressing mice proved to be ameliorated [65].

## 4. Aβ Neurotoxicity

In addition to NFTs, extracellular aggregates of Aβ peptide, called senile plaques (SPs), represent the most relevant histopathological hallmarks of AD [44].

Aβ peptide is generated through sequential proteolysis of the APP catalyzed by β- and γ-secretases. The first enzyme is an aspartyl protease, which is also known as β-site APP cleaving enzyme (BACE-1) and regulates the first and rate-limiting step of APP processing. Interestingly, a molecular interplay between Aβ and τ in causing synergic toxicity has been found, and GSK-3β has been recognized as the molecular linker between Aβ and τ. Indeed, the GSK-3β pathological activation by Aβ, by preventing the inhibitory phosphorylation of this PK, leads to an increase of τ phosphorylation, and GSK-3β inhibition decreases Aβ production and Aβ-induced neurotoxicity by reducing the BACE-1 cleavage of APP (Figure 6) [61]. Additionally, soluble oligomers of the Aβ peptide (AβOs) interfere with NMDA receptor (NMDAR) function, induce abnormal calcium influx and neuronal oxidative stress, and promote aberrant activation of GSK-3β [66].

DYRK1A phosphorylates APP at Thr668, enhancing its cleavage by both β- and γ-secretases [67], and increases the proteolytic activity of this latter enzyme by phosphorylation of its subunit called presenilin 1 (PS1) [68,69]. Likewise, the Aβ peptide has been reported to induce an increment of DYRK1A mRNA levels and to lead to τ phosphorylation at Thr212 under τ overexpression in neuroblastoma cells, suggesting DYRK1A as a key molecule bridging Aβ production and τ phosphorylation in AD (Figure 6) [70].

Lines of evidence have also documented the implication of FYN in Aβ-induced neuronal dysfunction and the existence of Aβ, τ, and FYN cooperation in AD-related pathogenesis [71]. Although the molecular mechanism underlying Aβ-mediated activation of FYN is still unclear, recent studies have demonstrated the formation of a ternary complex among soluble Aβ, the membrane-anchored protein known as cellular prion protein (PrP^c^), and FYN at the plasma membrane, resulting in τ missorting and hyperphosphorylation at Tyr18 (Figure 6) [23,71,72].

## 5. Nrf2 Signaling Pathway

Oxidative stress appears to be a major determinant of the pathogenesis and progression of different neurodegenerative diseases, including AD. Commonly, oxidative stress is caused by an imbalance between reactive radical species, among other reactive oxygen species (ROS), and a loss of function of many antioxidant defense enzymes, resulting in a disequilibrium between the formation of cellular oxidants and the antioxidative processes [73,74].

Nrf2 is one of the major regulators of cytoprotective responses to endogenous and exogenous stresses caused by ROS and electrophiles. In basal conditions, it is bound to its endogenous inhibitor Kelchlike ECH-associated protein 1 (Keap1), a cysteine-rich zinc-metalloprotein, that promotes Nrf2 degradation. In response to stress insults, such as ROS, this factor is released from Keap1, and upon translocation to the nucleus, it binds to the antioxidant response element (ARE), promoting the expression of some phase II detoxifying enzymes and antioxidant stress genes, namely NQO1, heme oxygenase-1, glutathione S-transferase, and aldo-keto reductase. Moreover, Nrf2 ameliorates the inflammation response by inhibiting the translocation of the nuclear factor-κB (NF-κB) and activating anti-inflammatory genes.

A linkage between GSK-3β/Nrf2 signaling pathway dysregulation and the reduction of oxidative stress defenses in both AD and PD has been demonstrated. Activated GSK-3β plays a pivotal role in the downregulation of Nrf2 through direct phosphorylation at Ser338 and Ser335 of Neh6 domain of Nrf2 and consequent proteasomal degradation of this latter in a Keap1-independent manner [36,75,76]. Notably, GSK-3β phosphorylation at two priming sites Ser342 and 347 by additional PKs including DYRK1A boosts GSK-3β activity toward Nrf2 (Figure 6) [76].

It is noteworthy that activated GSK-3β phosphorylates Src A subfamily members, including FYN, that enter the nucleus, leading to the phosphorylation of Nrf2 at Tyr568, nuclear export, and subsequent degradation of Nrf2 (Figure 6) [77,78,79,80,81,82].

## 6. α-syn Phosphorylation

PD is neuropathologically characterized by the presence of α-synuclein (α-syn)-containing Lewy bodies and loss of dopaminergic neurons in the substantia nigra, manifesting as reduced facilitation of voluntary movements. In this scenario, different investigations suggested that α-syn neurotoxicity in PD and related synucleinopathies may result from an imbalance between the detrimental oligomer-promoting effect of α-syn phosphorylation at Ser129 and the neuroprotective action of α-syn phosphorylation at Tyr125, which inhibits toxic oligomer formation [83,84].

In addition to τ protein, GSK-3β phosphorylates α-syn at Ser129. Interesting cooperation between α-syn and τ in increasing the magnitude or rate of phosphorylation of the other by GSK-3β has been demonstrated, establishing a novel upstream role for GSK-3β as one of several PKs associated with aberrant post-translational modifications (PTMs) of key proteins known to be causal in PD (Figure 6) [85].

Several in vitro and in vivo evidence have supported a potential neuroprotective activity of FYN due to its capability to phosphorylate α-syn at Tyr125 (Figure 6). However, it has been demonstrated in microglial cell lines treated with aggregated α-syn, which may induce FYN downregulation at the transcriptional level as a compensatory negative feedback loop, potentially “aiming” to protect the cell against FYN overactivation. Nevertheless, the final effects of FYN-mediated α-syn phosphorylation has yet to be elucidated [24,86].

DYRK1A also plays an important role in PD and additional synucleinopathies. Its capability to bind to α-syn and phosphorylate the same protein at the Ser87 residue facilitates intracellular inclusion formation (Figure 6) [87].

## 7. NMDAR-LTP and LTD Impairment

The activation of *N*-methyl-d-Aspartate receptors (NMDARs) has been recently implicated in AD and related to synaptic dysfunction. While synaptic NMDARs are neuroprotective, overactivation of those located outside of the synapse cause a loss of mitochondrial membrane potential and cell death. 

Most native NMDARs are heterotetramers containing two glycine-binding NR1 and two glutamate-binding NR2 subunits, and the majority ones comprise the obligatory subunit GluN1 plus either GluN2B or GluN2A or a mixture of the two. NMDARs are the primary channel that mediates Ca^2+^ signals in hippocampal neurons and contribute to the expression of long-term potentiation (LTP) and long-term depression (LTD), which are two major forms of long-lasting synaptic plasticity, by employing both NR2A and NR2B subunits [88]. LTP is characterized by increased synaptic efficacy and is thought to be one of the neurophysiological process correlates of learning and memory.

GSK-3β has been firmly established as a key player in synaptic plasticity, since its activity blocks NMDAR-LTP and induces NMDAR-LTD (Figure 6) [89]. In detail, it has been demonstrated that during LTD, GSK-3β activity is increased as a result of its dephosphorylation at Ser9 by phosphatase PP1; whereas, during LTP, the activation of NMDARs leads to stimulation of the PI3K-Akt pathway, which inhibits GSK-3β by Ser9 phosphorylation [90,91].

Several pieces of evidence supported an upstream regulator role for FYN on NMDA receptors [23] and the involvement of the same non-receptor tyrosine kinase in LTP [92]. Activated FYN phosphorylates both NR2A and NR2B subunits of the NMDARs, selectively elevates NR2B trafficking and membrane stabilization, resulting in an increment of synaptic expression and receptor transmission [93]. Three major Tyr residues in the GluN2B *C*-terminal tail of NMDARs have been identified as FYN phosphorylation sites: Tyr1252, Tyr1336, and Tyr1472, among which the latter is the most prominently phosphorylated site in vitro (Figure 6). Moreover, the phosphorylation of GluN2 subunits by exogenous FYN is dependent on its binding to the postsynaptic density (PSD) proteins 93 and 95 (PSD-93 and -95) [94].

DYRK1A can also regulate neural development and synaptic plasticity through the phosphorylation of the NMDARs subunit GluN2A at Ser1048 (Figure 6) [95].

## 8. Neuromuscular Alterations in MND

Motor neuron disease (MND) represents a wide and heterogeneous group of neuromuscular disorders, including ALS, SMA, and DM1, resulting in the loss of motor neurons and progressive muscle wasting. The most common hereditary forms of SMA are caused by large deletions that inactivate the SMN1 gene, leading to low levels of the ubiquitously expressed protein survival of motor neuron (SMN) with a predominant function in neuronal development and synapse formation [21,96]. In skeletal muscle, GSK-3β is a negative regulator of growth through dysregulation of the myogenic regulator factors. Indeed, GSK-3β inhibition in C2C12 myoblasts and C57BL/6 mice enhanced the myogenic regulator factor activity, myotube formation, and muscle growth [96]. Moreover, potent and reasonably selective GSK-3β inhibition proved to prolong the median survival of a transgenic Δ7 SMA KO mouse model of SMA and showed neuroprotective effects in a cell-based SMA-related model of oxidative stress-induced neurodegeneration [21]. Increased expression and activity of GSK-3β have also been reported in the skeletal muscle of patients with DM1, which is a complex disease linked to the reduction of cyclin D3 due to its phosphorylation at Thr283 by active GSK-3β (Figure 6) [20,97].

## 9. GSK-3β Modulation

**Figure 7:**
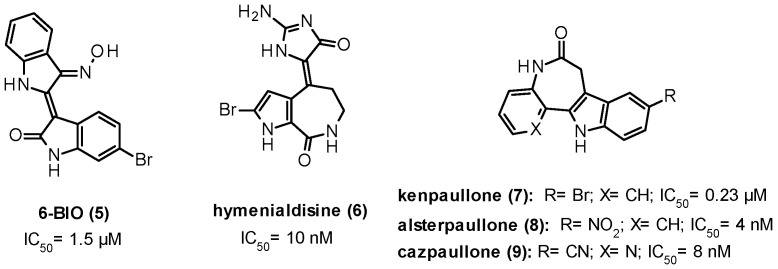
Different chemical classes of ATP-competitive GSK-3β inhibitors endowed with promising CNS preclinical potential.

Over the last two decades, the increased interest in GSK-3β led to the discovery of many inhibitors based on chemically different molecular scaffolds and acting with diverse mechanisms of action namely ATP and non-ATP competition, and allosteric modulation [98]. Most inhibitors reported in the literature are ATP competitive agents; some of them have synthetic origin, whereas others have been derived directly or indirectly from small molecules of natural origin (e.g., paullones, maleimides, indirubins, arylindolemaleimides, thiazoles). Several of these GSK-3β inhibitors have been evaluated in preclinical studies (e.g., 6-bromoindirubin-3′-oxime (6-BIO, 5), hymenialdisine (6), kenpaullone (7), alsterpaullone (8), cazpaullone (9), and SB216763 (10), Figure 7 and Table 1), and some of them showed promising CNS-related preclinical data, namely neuroprotection, decrease of τ phosphorylation, therapeutic benefits in AD, and schizophrenic models. Among them, the arylindolemaleimide 10 (Table 1) is a highly selective nanomolar GSK-3 inhibitor developed by GlaxoSmithKline that showed neuroprotective effects against a variety of pro-apoptotic conditions, including inhibition of the PI3 kinase/Akt survival pathway, trophic deprivation, Aβ toxicity, heat shock, ethanol, NMDA excitotoxicity, and polyglutamine toxicity caused by the HD protein. Interestingly, in an AD model of mice injected with Aβ peptide, 10 reduced Aβ neurotoxic effects, including reduction in τ phosphorylation, caspase-3, and the activity of the stress-activated kinase JNK (c-Jun *N*-terminal kinase). However, the same inhibitor produced neurodegenerative-like effects and behavior deficits in healthy mice, suggesting how over-inhibition of GSK-3 may result in conditions that prevent neurons from operating normally [19].

AZD1080 (11, Table 1) has been reported by AstraZeneca as a potent, orally active and brain permeable GSK-3 inhibitor, which proved to inhibit both recombinant human GSK-3α and GSK-3β in the nanomolar range (K_i_ = 6.9 nM and 31 nM, respectively) and showed selectivity toward CDK2 (K_i_ = 1150 nM; 37-fold), CDK5 (K_i_ = 429 nM; 14-fold), CDK1 (K_i_ = 1980 nM; 64-fold), and Erk2 (K_i_ > 10 µM; >323-fold), as well as 23 different kinases and 65 diverse receptors, enzymes, and ion channels. Notably, sub-chronic treatment with 11 prevented disruption of LTP induction caused by an acute challenge with MK-801, an NMDA blocker, suggesting a protective effect of this inhibitor in dysfunctional systems. Additionally, the same compound was able to reduce τ phosphorylation at Ser396 in 3T3 fibroblasts engineered to stably express 4-repeat human τ protein (IC_50_ = 324 nM) [91]. The high permeability (8 × 10^−3^ cm min^−1^) of 11 predicted using an in vitro bovine endothelial cell assay suggested a significant brain exposure in vivo. The same derivative was in phase I clinical trial targeting AD; unfortunately, further development of this inhibitor was halted due to the observed nephrotoxicity [99].

One of the main limitations for the therapeutic use of ATP-competitive inhibitors of PKs is the lack of kinase selectivity due to the high homology degree of their catalytic sites. In this respect, the involvement of the GSK-3β in essential molecular pathways (e.g., the oncogenic β-catenin signaling) suggested the potential risks associated to GSK-3 inhibition in a chronic treatment and the need for developing selective subtle modulators to offer a safer homeostasis recovery effect without interfering in other cellular signaling [100]. Therefore, several efforts have been devoted to developing different chemical families of non-ATP competitive GSK-3β inhibitors able to bind unique regions of the enzyme and act by various mechanisms of action: covalent inhibition, modulation of key residues in the GSK-3β active site, substrate competitive inhibition, and binding of the ribose region of the ATP site [19].

### 9.1. Covalent Inhibitors

Halomethylketone (HMK) derivatives have been described as the first GSK-3β irreversible inhibitors. They can form an irreversible covalent sulfur–carbon bond between the critical Cys199, which is located at the entrance to the ATP site, and their HMK moiety. Since Cys199 in this PK is not conserved in other structurally related kinases, such as CDK-1, CDK-2, or CDK-5, covalent modification of this key residue could offer promises to achieve specificity. In this scenario, Perez et al., aimed at developing useful pharmacological tools to explore physiological and pathological processes related to GSK-3β, designed and synthesized novel phenylhalomethylketones as bioisosters of the irreversible inhibitors previously reported [101,102]. The authors confirmed the essential role of the halomethylketone (HMK) moiety for the enzyme inhibition, since the replacement of the halide atom in the α position of the carbonyl moiety and/or the carbonyl group substitution with an oxime function produced detrimental effects on the affinity. Among all derivatives, analogs 12–14 (Table 1) proved to be low micromolar inhibitors (IC_50_ = 2.5 µM for derivatives 12 and 14, and 0.5 µM for compound 13) and at 10 µM showed GSK-3β selectivity versus several PKs, namely Abl-K, EGFR-K, IR-K, MAP-K, MEK-1 K, PK p56, and Src-K and neurotransmitter receptors (e.g., hD2 and hD3, NMDA, AMPA, α2, 5-HT, etc.). Interestingly, in a chemical reactivity study, in which UPLC-MS was employed to detect the formation of S-adducts, derivative 12 displayed high susceptibility to react with thiol groups, and an enhancement of the compound reactivity upon addition of a suitable base as trimethylamine was observed. Moreover, analog 14 showed cell permeability and at 25 µM concentration, similarly to lithium chloride, proved to interfere with GSK-3β-mediated phosphorylation of PHF-1, which is an epitope specifically phosphorylated at Ser396 by the enzyme on the τ protein after 16 h of treatment [103].

Yang et al. developed a series of (aza)indolyl maleimide covalent inhibitors by utilizing mild reactive groups such as acrylamido or α-fluoroacetamido onto the maleimide scaffold. Among all derivatives, compound 15 (Table 1) showed nanomolar potency against the enzyme (IC_50_ = 17 nM) and high GSK-3β selectivity versus TAK1 and CDK2 (IC_50_ = 2753 nM and 639 nM, respectively). In addition, the same inhibitor in human SH-SY5Y neuroblastoma cells reduced τ phosphorylation at Ser396 in a dose-dependent manner and in hMDRI-MDCK cells proved to permeate the BBB (Papp = 41.9 × 10^−6^ cm s^−1^) and to not be a P-gp substrate [104].

The small heterocyclic thiadiazolidindiones (TDZDs) represent the first non-ATP competitive GSK-3 inhibitors reported by Martinez et al. in 2002 as novel disease-modifying agents with both good selectivity and excellent therapeutic effects on neurodegenerative disorders associated with τ hyperphosphorylation. Although the exact mechanism of action of these inhibitors has not yet been experimentally confirmed, an irreversible interaction with Cys199 of GSK-3β has been recognized at the basis of their enzyme inhibition [105]. Within this group, Tideglusib (16, Table 1), a brain-permeable irreversible nanomolar inhibitor of GSK-3β (IC_50_ = 50 nM), was able to decrease τ phosphorylation and reduce brain amyloid plaques in preclinical models [106,107]. The same inhibitor proved to not have safety concerns in phase II clinical trials for AD and progressive supranuclear palsy (PSP), a rare tauopathy, after long-term treatments of 12 and 6 months, respectively [108,109]. Since participants did not show improvement on either of the primary outcome measures and some secondary exploratory endpoints, further drug development was stopped for both two diseases [109,110].

### 9.2. Substrate Competitive Inhibitors

Substrate competitive inhibitors (SCIs) represent a different class of GSK-3β modulators able to engage the substrate domain of GSK-3β, which is a less conserved binding site with a unique folding different from other PKs. Although the great potential of these agents in terms of selectivity and specificity has been recognized, they have not been so extensively investigated. 

Palomo et al. developed novel 5-imino-1,2,4-thiadiazoles (ITDZs) as brain-permeable SCI small compounds to potentially modify the neurodegeneration course by decreasing neuronal injury and repairing the damaged brain. The majority of these ITDZs showed GSK-3β inhibition in the low-submicromolar range. Among them, 17–21 (Table 1) were selected to assess their capability to affect the production of nitrites from primary cultured glial cells, astrocytes, and microglia after treatment with LPS and to protect neurons from the injury induced by the cell-free supernatant from LPS-activated microglia, which is a cellular model for the damage caused by brain environment in neurodegenerative diseases. Remarkably, all three derivatives decreased the nitrile production emerging as promising anti-inflammatory and neuroprotective agents and displayed the ability to differentiate neural stem cells to mature neurons [111].

Liang and Li developed selective, substrate-competitive, and passive membrane permeable GSK-3β inhibitors based on the 6-C-glycosylflavone isoorientin (22, Table 1) as valuable chemical probes and drug leads with therapeutic potential to tackle AD and other GSK-3β relevant diseases. Among these inhibitors, 23 (Table 1) showed brain permeability (Pe = 2.23 × 10^−6^ cm·s^−1^, parallel artificial membrane permeability assay (PAMPA)-BBB), submicromolar inhibitory potency against GSK-3β (IC_50_ = 0.59 µM), and effectively attenuated τ hyperphosphorylation at Ser 396 in a dose-dependent manner in an in vitro assay using a whole-cell lysate of human SHSY5Y neuroblastomas. Moreover, 23 at 5 μM showed a good selectivity as it effectively inhibited GSK-3β by decreasing 92.3% kinase activity compared to the control (100% kinase activity). In contrast, only marginal or weak inhibition against 40 out of 41 PKs relevant for AD and other CNS disorders was observed. In a cellular model of AD where Aβ_42_ oligomers were administrated in human SH-SY5Y neuroblastomas, 23 displayed a good tolerability profile similar to 22, as no cytotoxicity up to 1000 μM dose was observed. Moreover, pretreatment of SH-SY5Y cells with 23 (1.25–20 μM) for 1 h followed by coincubation with 10 μM Aβ_42_ for 72 h recovered cell viability from 40% to 100% in a dose-dependent manner. The neuroprotective potency of compound 23 (EC_50_ = 8.7 μM) was 5.4-fold higher compared to that of 22 (EC_50_ = 47 μM) [112]. 

In 2020, Rippin et al. developed a novel series of GSK-3 SCI compounds as promising leads for future drug development. The authors took advantage of their previously described structural models of GSK-3 bound to short phosphorylated SCI peptides, including a phosphorylated residue (usually Ser) in the context of SXXXS(p) (where S is the target Ser, S(p) is phosphorylated Ser, and X is any amino acid) [113,114,115]. Among all derivatives, 24 and 25 (Table 1) were identified as the most potent inhibitors showing IC_50_ values of ≈1–4 µM. Both compounds exhibited a similar affinity for GSK-3α due to the high similarity of its substrate’s binding site with that of GSK-3β, and analog 25 demonstrated GSK-3 selectivity over a panel of 30 PKs. Remarkably, the same compound proved to inhibit GSK-3β in human neuroblastoma SH-SY5Y cells at 1–5 µM concentrations and reduce τ phosphorylation at Ser396 in mouse hippocampal primary neurons at 20 µM [115]. 

**Table 1:** GSK-3β inhibitors and their applications.

Chemical Structure	pK_i_/IC_50_ Values	Purpose/Biological Activities
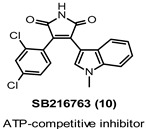	IC_50_ 34.3 nM	Neuroprotection against Aβ and NMDA excitotoxicity. Decrease of τ phosphorylation [19].
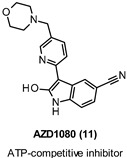	K_i_ 31 nM	Rescue of the synaptic plasticity deficits. Inhibition of τ phosphorylation at Ser396 in 3T3 fibroblasts (IC_50_ = 324 nM). Phase I clinical trial targeting AD [91,99].
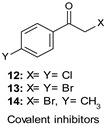	IC_50_ 0.5–2.5 µM	Pharmacological tools [101,103].
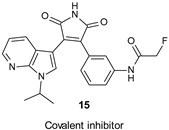	IC_50_ 17 nM	Reduction of τ phosphorylation at Ser396 in human SH-SY5Y cells [104].
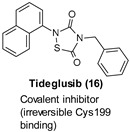	IC_50_ 50 nM	Decrease of τ phosphorylation and reduction of brain amyloid plaques. Phase II clinical trials for AD and PSP [99,106,108,109,110].
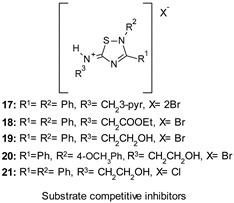	IC_50_ 0.6–7 µM	Decrease of nitrites production from primary cultured glial cells, astrocytes, and microglia after LPS treatment. Ability to differentiate neural stem cells to mature neurons [111].
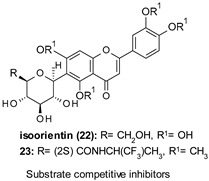	22: IC_50_ 184.9 µM 23: IC_50_ 0.59 µM	Attenuation of τ hyperphosphorylation at S396. Neuroprotective activity against Aβ_42_ toxicity in SH-SY5Y cells (EC_50_ = 8.7 µM) [112].
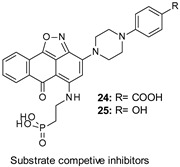	IC_50_ ≈ 1–4 µM	GSK-3β inhibition in human neuroblastoma SH-SY5Y cells. Reduction of τ phosphorylation at Ser396 in mouse hippocampal primary neurons [115].
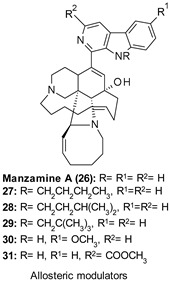	26: IC_50_ 1.5 µM	Decrease of τ phosphorylation in cell cultures. Anti-neuroinflammatory properties by reducing the phorbol 12-myristate 13-acetate-stimulated generation of O^2−^ and TXB2 from activated rat neonatal microglia (IC_50_ = 0.03–0.4 µM) [32,116,117].
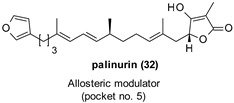	IC_50_ 1.9 µM	Chemical tool for GSK-3β-mediated diseases [118].
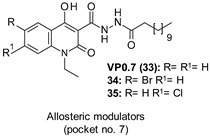	IC_50_ 2.01–3.01 µM	Anti-inflammatory properties in cellular models of neurodegenerative diseases. Decrease of nitrite release after LPS stimulation. Efficacy in preclinical models of multiple sclerosis and fragile X syndrome. Improvement of delayed myogenesis in CDM1 myoblasts and neuroprotection properties in SMA-derived cells [20,32,119,120,121].

### 9.3. Non-ATP Competitive Modulators

Different marine sponges have represented a source of natural products (NPs) active against neurological disorders. Among them, manzamine A (26, Table 1), characterized by a unique 5-, 6-, 6-, 8-, 13-membered heterocyclic ring system coupled to a β-carboline moiety, emerged as cell-permeable GSK-3 inhibitor able to decrease τ phosphorylation in cell cultures. Moreover, inhibition studies of 24 against five different GSK-3β related kinases, including CDK-1, PKA, CDK-5, MAPK, and GSK-3α confirmed the compound specificity in inhibiting GSK-3β and CDK-5, suggesting manzamine framework as a promising scaffold for developing more potent and selective GSK-3β/CDK5 as anti-AD agents. In SAR exploration studies, Peng et al. described the promising anti-neuroinflammatory properties of 26 and some analogs 27–31 (Table 1) in terms of the capability of reducing the phorbol 12-myristate 13-acetate-stimulated generation of superoxide anion (O_2_^−^) and thromboxane B2 (TXB2) from activated rat neonatal microglia (IC_50_ = 0.03–0.4 µM). Further molecular modeling studies revealed a potential allosteric site on GSK-3 for these inhibitors corresponding with the “phosphate-binding pocket” (Arg96, Arg180, and Lys205), near the activation site and proposed to be the binding pocket for TDZDs by Martinez et al. [32,105,116,117].

Bidon-Chanal et al. reported the isolation and biochemical characterization of the sesquiterpene palinurin (32, Table 1), an NP extracted from the marine sponge *Ircinia variabilis*, as the first non-ATP/substrate modulator of GSK-3β able to bind to an allosteric site at the *N*-terminal lobe of the enzyme, which was previously discovered as pocket no. 5. Interestingly, MD simulations confirmed a novel allosteric mechanism of action for this compound based on the modulation of the accessibility of the ATP γ-phosphate of the enzyme by constraining the conformation of its glycine-rich loop. The same mechanism was recognized responsible for conferring to 32 a high degree of GSK-3β (IC_50_ = 1.9 µM) selectivity over different kinases (e.g., CDK-5: IC_50_ > 25 µM; CDK1, MAPK, and CK2: IC_50_ > 100 µM). In the light of these promising results, 32 was identified as a promising candidate for the development of new selective and more potent drugs for the treatment of GSK-3β-mediated diseases [118]. 

Within the allosteric modulation context, in 2011, Palomo et al., during an in vitro screening activity assay on GSK-3β of their in-house chemical library, identified the quinoline derivative VP0.7 (33, Table 1) as an interesting low micromolar inhibitor (IC_50_ = 3.01 μM) able to bind an allosteric site on the enzyme according to the results of different kinetic experiments. Docking studies considering the whole protein surface allowed the authors to recognize pocket no. 7 as the allosteric binding site of 33 and hypothesize a change in the activation loop of GSK-3β as responsible for the allosteric modulation of the enzyme [32]. The same compound, along with different derivatives, was patented by Martinez et al. as a low micromolar allosteric GSK-3β inhibitor (IC_50_ = 2.85 µM) with promising anti-inflammatory properties in cellular models to treat neurodegenerative and inflammatory diseases [119]. Remarkably, 33 at various concentrations (1.25, 2.5, 5, and 10 µM) showed a dose-dependent effect on the decrease of nitrite release after LPS stimulation. In further studies, the same quinolone derivative showed safety and great efficacy in preclinical models of multiple sclerosis and fragile X syndrome, pointing to potential use in the chronic treatment of such neurological diseases [120,121]. 

In 2017, Palomo et al., considering the emergent role of GSK-3β in neuromuscular degenerative diseases, designed and synthesized novel quinoline-3-carbohydrazide-based compounds as specific GSK-3β inhibitors to treat chronic diseases such as congenital myotonic dystrophy type 1 (CDM1) and SMA. Several efforts were directed at developing selective subtle modulators of GSK-3β to provide a safe enzyme homeostasis recovery effect without interfering in the oncogenic β-catenin signaling. Among all compounds, two halogen derivatives of 33, 34 and 35 (Table 1) with low micromolar inhibitory activity against GSK-3β (IC_50_ = 2.01 and 2.48 µM, respectively) showed a selectivity profile and a mechanism of action similar to those of 33. In detail, both analogs displayed a consistent and robust selectivity versus a panel of 50 protein kinases at a fixed concentration of 10 µM, and in molecular modeling studies induced a conformational change in the GSK-3β active site by modification of the enzyme activation loop flexibility. 

A challenge for potent GSK-3 (α- or β-) inhibitors is the reduction of the abnormal enzyme activity while not promoting oncogenesis through aberrant β-catenin signaling. GSK-3 (α- and β-isoforms) mediates a phosphorylation event that retains low the β-catenin levels by promoting its ubiquitylation and proteosomal degradation. When this phosphorylation event is blocked, β-catenin is stabilized and accumulated in the cytosol and translocates to the nucleus, where it coactivates the transcription of different oncogenes. To corroborate the great potential of allosteric modulators in overcoming the great challenge in GSK-3β targeting, the effect of 33 and 34 on β-catenin localization was evaluated in two different human cell lines of glioblastoma and neuroblastoma, LN-18 and SH-SY5Y, respectively. Encouragingly, in cells cultured for 72 h in the presence of both inhibitors at a concentration of 10 μM immunofluorescence analysis of subcellular distribution of β-catenin revealed the localization of β-catenin in the cytosol. Moreover, in human samples from patients with CDM1 and SMA, quinoline 34 improved delayed myogenesis in primary myoblasts from skeletal muscle of patients with CDM1 and, as well as 33, proved to have neuroprotective properties in SMA-derived cells [20].

## 10. FYN Inhibition

Given the above-mentioned crucial role of FYN in the CNS, the development of FYN-targeted agents could offer promises to achieve effective therapies for neurodegenerative diseases. FYN modulation could be accomplished by interaction with different binding sites on specific enzyme domains. Since SH2 and SH3 domains interact with target proteins, chemical entities able to disrupt that protein–protein interaction could regulate the kinase activity. However, most FYN inhibitors reported so far are ATP-competitive agents able to interact with crucial residues of the enzyme catalytic site [43]. Unfortunately, due to the strict similarity of most SFKs in their catalytic domains, no compounds selective for FYN have been reported. Indeed, all the published inhibitors showed inhibitory potency toward other members of SFKs or some TKs. Moreover, because of the high degree of similarity of the FYNB and FYNT isoforms’ catalytic domain [122], some FYN inhibitors developed as non-CNS agents, such as 1 and 2 (Table 2), have been repurposed to treat CNS disorders. It is worth mentioning that the lack of selectivity combined with the high potency of most ATP-competitive FYN inhibitors requires intensive efforts and new strategies including a multi-target approach to developing agents with a higher safety profile to avoid the potential interference with relevant physiological pathways, such as myelination. In this context, it would be necessary again to take into consideration a selective subtle modulation of FYN kinase.

Compound 1 (AZD0530) is an SFKs inhibitor based on a quinazoline heterocycle (Table 2) and able to inhibit Src, FYN, Yes, and Lyn with IC_50_ values ranging from 2 to 10 nM [13]. Although limited benefits in phase II clinical studies were observed as an anti-cancer agent, the excellent pharmacokinetic properties (oral bioavailability >90%; half-life of approximately 40 h [14]) combined with the good BBB permeability encouraged 1 repurposing as a CNS agent. Recent studies supported the potential employment of 1 in tauopathies treatment due to its ability to reduce τ hyperphosphorylation. Yadikar et al. evaluated in a tauopathy cell-based model the effect of different PKs inhibitors, including 1, which displayed a substantial reduction of τ phosphorylated in both monomeric (40%) and oligomeric (46–75%) forms [123]. 

Tang et al. also studied the compound efficacy in PS19 transgenic mice and traumatic tauopathy models. After oral administration (5 mg/kg/d) for 9 months, 1 crossed the BBB, inhibited FYN, and reduced τ phosphorylation in mice brain without altering both proteins expression. As a result, an improvement of the mice cognitive functions was observed. Notably, in an additional investigation, the same authors revealed no change in pTyr18 τ levels in PS19 mice treated with 1 (5 mg/kg/d) for 7 months. However, the reduction of τ phosphorylation at Ser202, Ser396, Ser404, and Thr205 was observed, suggesting a suspension of FYN-mediated mislocalization of τ to the post-synaptic area and prevention of further τ spreading between neurons as a result of 1 binding to FYN [124]. A phase Ib study was also launched in 2013 to assess the safety and tolerability of 1 in mild-to-moderate AD patients with a daily oral dosing of 100–125 mg during 4 weeks. Compound 1 proved to be in general safe, well-tolerated, and reached good levels in cerebrospinal fluids, although no beneficial effect was observed on measures of cognitive and neuropsychiatric function, activities of daily living, or cerebral glucose metabolism [125]. A further phase IIa study finished in 2018 confirmed a not-relevant effect on cerebral metabolic rate of glucose in AD patients after 52 weeks of treatment [126]. An early phase I study to explore the effect of 1 on brain activity associated with visual processing in patients with PD psychosis is still ongoing (NCT03661125) [127]. 

Derivative 2 is a phenylaminothiazole derivative (Table 2) first developed as a C-Kit receptor inhibitor for the treatment of tumors in animals and currently under evaluation to treat human cancer. The same compound has been also investigated as a potential agent for treating inflammatory diseases such as rheumatoid arthritis. In the context of CNS therapies, 2 has been repurposed as a promising anti-AD agent able to reduce τ hyperphosphorylation and prevent NFTs formation, taking into account its nanomolar inhibitory potency against FYN (IC_50_ = 240 nM) [23]. In a phase II study, 2 was administered twice a day (3 or 6 mg/kg/d) with a cholinesterase inhibitor and/or memantine (an NMDA antagonist) for 24 weeks to mild-to-moderate AD patients [128]. A relevant slower cognitive decline was observed in patients treated with the FYN inhibitor compared with the placebo group providing evidence for the great potential of 2 as an AD-modifying agent. Moreover, in an ongoing phase III study, the safety and efficacy of 2 for the treatment of mild to moderate AD are under investigation [15].

With the aim to discover new selective FYN inhibitors as promising drug candidates or useful tools for studying the complex biological pathways modulated by FYN in CNS, several virtual screening campaigns have been carried out. Poli et al., combining a Fingerprints for Ligands and Proteins (FLAP) ligand-based similarity analysis with docking and MD simulations, identified a few hit compounds endowed with low micromolar inhibitory potency against FYN from a commercially available Asinex library of 305,625 chemical entities. Among them, compound 36 (Table 2), proved to be the most potent FYN inhibitor (IC_50_ =  4.8 μM) and was selected for docking simulations to elucidate the crucial binding interactions of its 3-amino-1,2,4-triazin-5(2*H*)-one scaffold at the ATP binding pocket of FYN. A preliminary hit-to-lead optimization campaign allowed identifying a new derivative (37, Table 2) six-fold more active (IC_50_ = 0.76 μM) than compound 36 [46,129].

**Figure 8:**
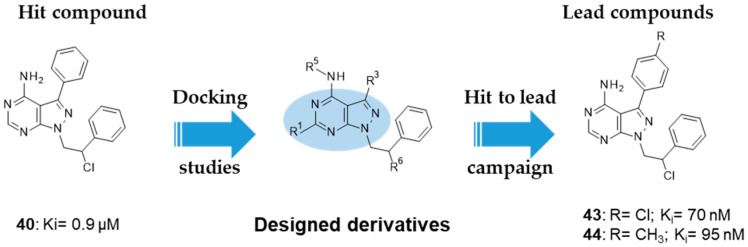
Schematic representation of the development of lead FYN inhibitors 43 and 44 starting from hit compound 40 (left). Chemical structures of FYN inhibitors 41 and 42 employed in the docking studies during a hit-to-lead optimization campaign [130].

Tintori et al. identified by a virtual screening three micromolar ATP-competitive FYN inhibitors (38–40, Table 2) as promising hit compounds for both tauopathies and cancer treatment (K_i_ = 2.1, 2.25 and 0.9 µM, respectively). Starting from hit 40, a racemic compound bearing a pyrazolo[3,4-*d*]pyrimidine core structurally related with that of well-known non-selective Src inhibitors PP1 (41) and PP2 (42, Figure 8), a hit-to-lead optimization campaign was carried out allowing the identification of analogs 43 and 44 (both racemates, Table 2) as the most potent FYN inhibitors (K_i_ = 70 and 95 nM, respectively). 

Among the latter ones, derivative 43 did not significantly inhibit at 10 µM any of the Ser/Thr kinases tested (PIM-1, mTOR, JNK, CDK5, CHL1), showing selectivity for Src family members over Ser/Thr kinases, including DYRK1A and GSK-3β, which were also implicated in AD pathology. Further studies evidenced the ability of both 43 and 44 to reduce Tyr18-τ phosphorylation mediated by FYN. In detail, in SH-SY5Y cells treated with Aβ 1–42 (Aβ42) oligomer/protofibril to induce AD-like neurotoxicity, both compounds reduced τ phosphorylation at Tyr18 in a dose-dependent manner. Although solubility issues were observed for both 43 and 44 inhibitors in ADME in vitro studies, good values of metabolic stability in human liver microsomes, passive membrane permeability, and BBB permeability in PAMPA assays suggested the great potential of these derivatives as promising agents to tackle tauopathies such as AD [130].

**Figure 9:**
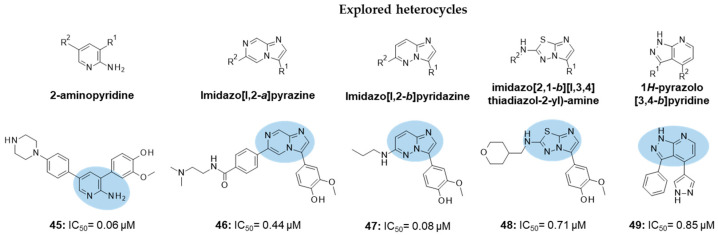
Explored heterocycles (**top**) and representative examples (**bottom**) of FYN inhibitors developed by Lau [131].

Lau developed new FYN inhibitors with potential therapeutic application in several diseases including AD and PD within the CNS space. Among them, the inventor mainly claimed derivatives based on 3,5-disubtituted 2-amino pyridine, 3,6-disubtituted imidazo[l,2-*a*]pyrazine, 3,6-disubtituted imidazo[l,2-*b*]pyridazine, *N*- and 5-disubtituted imidazo[2,1-*b*][l,3,4]thiadiazol-2-yl)-amine, and 3,4-disubstituted 1*H*-pyrazolo[3,4-*b*]pyridine heterocycles (Figure 9). Extensive decoration with moieties of different chemical nature (e.g., alkyl, heterocyclyl, aryl or heteroaryl substituents) were explored for all these different heterocycles exemplified in compounds 45–49 (Figure 9 and Table 2), which showed submicromolar inhibitory potency against FYN [131].

**Figure 10:**
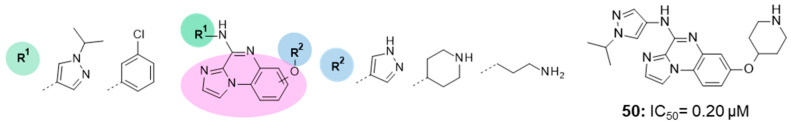
Fyn inhibitors based on 4-amine-imidazo[1,2-*a*]quinoxalin scaffold (magenta) exploring different 4-amino (green) and 6 or 7-ethyl (cyan) substituents by Paraselli et al. [132].

Likewise, Paraselli et al. presented new small molecules based on 4-amine-imidazo[1,2-*a*]quinoxalin scaffold and bearing different substituents (Figure 10) as FYN inhibitors for PD treatment. The inventors explored different ether substituents at 6 and 7 positions of the imidazo[1,2-*a*]quinoxalin core (Figure 10, cyan) such as pyrazole and piperidine heterocycles or 3-aminopropyl alkyl chain. Furthermore, different aryl or heteroaryl substituents namely 3-chlorophenyl or *N*-isopropyl-4-pyrazoyl were also installed on the 4 amino group. Among all the derivatives described, compound 50 (Figure 10 and Table 2) has been selected as a representative inhibitor of this series that endowed high nanomolar activity against FYN kinase [132].

**Table 2:** ATP-competitive FYN inhibitors and their applications.

Chemical Structure	K_i_/IC_50_ Values	Purpose/Biological Activities
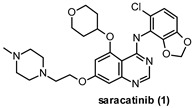	IC_50_ 2 nM	Reduction of τ phosphorylation in tauopathy cell-based model. Reduction of τ phosphorylation and NTFs formation in transgenic and traumatic tauopathy. Phases Ib and IIa studied in AD patients. Ongoing phase I study in patients with PD psychosis (NCT01872598) [13,72,127].
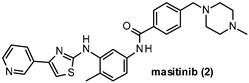	IC_50_ 240 nM	Slow cognitive decline in placebo-controlled phase II study in AD patients. Ongoing phase III study to assess the safety and efficacy in mild to moderate AD patients [23,128,133].
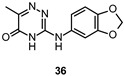	IC_50_ 4.8 μM	Hit compound [46].
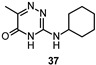	IC_50_ 0.76 μM	Lead compound [46].
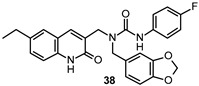	K_i_ 2.1 μM	Hit compound [130].
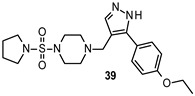	K_i_ 2.25 μM	Hit compound [130].
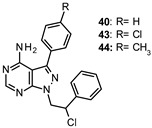	40: K_i_ 0.9 µM 43: K_i_ 70 nM 44: K_i_ 95 nM	Reduction of τ phosphorylation at Tyr18 in SH-SY5Y cells treated with Aβ 1–42 (Aβ42) oligomer/protofibril to induce AD-like neurotoxicity [130].
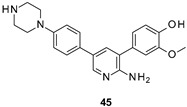	IC_50_ 60 nM	Potential application in AD and other tauopathies WO2017/044623 [131].
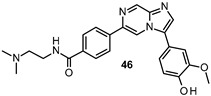	IC_50_ 0.44 μM	Potential application in AD and other tauopathies WO2017/044623 [131].
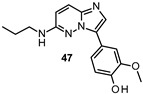	IC_50_ 80 nM	Potential application in AD and other tauopathies WO2017/044623 [131].
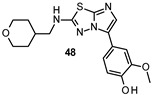	IC_50_ 0.71 μM	Potential application in AD and other tauopathies WO2017/044623 [131].
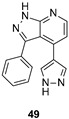	IC_50_ 0.85 μM	Potential application in AD and other tauopathies WO2017/044623 [131].
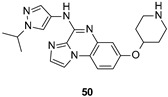	IC_50_ 0.20 μM	Potential application in AD and other tauopathies WO2017/037604 [132].

## 11. DYRK1A Inhibitors

DYRK1A involvement in cognitive deficits associated with various neurodegenerative disorders brought medicinal chemists to a long-standing interest in developing selective DYRK1A inhibitors to treat complex and unmet medical needs. 

**Figure 11:**
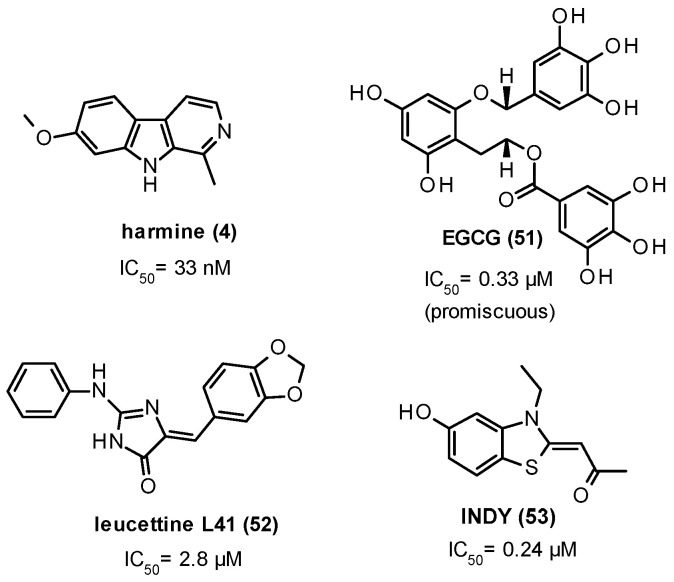
Chemical structures of major representatives of DYRK1A inhibitors.

Different NPs such as compound 4 [134], epigallocatechin-gallate (EGCG, 51) [135], and meridianins [136] and synthetic derivatives like leucettine L41 (52) [137] and INDY (53) [51] have been extensively explored within the DYRK1A inhibition space (Figure 11). 

These structurally unrelated compounds were remarkably reported, first by Pathak et al. [138] and afterward by Arbones et al. [25], as major representatives of DYRK1A inhibitors with nanomolar potency and significant cross-reactivity toward additional members of the DYRK family and other phylogenetically similar PKs, such as GSK-3, CLKs, and CDKs. Several optimization campaigns have been carried out from these representative inhibitors aimed at developing novel agents endowed with improved inhibitory potency and selectivity for DYRK1A as described in a recent patent overview by Nguyen et al. [139].

Frost et al. demonstrated the capability of 4 (Figure 11, IC_50_ = 33 nM), which is one of the most potent and selective ATP-competitive DYRK1A inhibitors presently available [140,141,142], to consistently interfere with τ protein phosphorylation. On one side, this nanomolar inhibitor proved to significantly reduce the DYRK1A-dependent phosphorylation at Ser396, Ser262/Ser356 (12E8 epitope), and Thr231 in both H4-tau cells at 0.8 µM and 8 µM and in vitro assays; on the other side, it blocked the direct phosphorylation of τ protein by DYRK1A on Ser396 with an IC_50_ value of 0.7 µM [134].

The employment of β-carboline alkaloids as 4 and derivatives as CNS tools has been hampered by their high affinity for multiple targets, namely the 5-hydroxytryptamine receptor substypes 5-HT2 and 5-HT1A, the NMDA receptor, monoamine oxidase (MAO-A) [134,143], and dopaminergic signaling pathways. Therefore, improved 4 analogs have been developed to overcome these limitations. Among them, AnnH75 (54, Table 3), maintaining a nanomolar potency against DYRK1A (IC_50_ = 181 nM), displayed a low affinity for MAO-A (IC_50_ > 10,000 nM), therefore resulting in less side effects in comparison with the parent compound. Moreover, 54 inhibited Tyr autophosphorylation of DYRK1A during translation at concentrations >1 μM and inhibited DYRK1A activity with an IC_50_ of 1 μM in cellular assays [144].

Compound 51 (Figure 11) is one of the main polyphenolic constituents of green tea. It proved to inhibit DYRK1A with IC_50_ = 0.33 µM in a non-ATP competitive manner and did not show DYRK1A selectivity over a vast panel of PKs, as reported by Bain et al. [145]. Remarkably, in a double-blind, randomized, placebo-controlled phase II trial (TESDAD), administration of a green tea extract containing 45% 51 in DS patients (600 mg/day in participants weighing 50–75 kg and 800 mg/day in participants weighing 75–100 kg) was significantly more effective than placebo and cognitive training at improving visual recognition memory, inhibitory control, and adaptive behavior [146].

Meridianin derivatives are marine alkaloids isolated from the south Atlantic tunicate Aplidium meridianum possessing kinase inhibitory activity [147,148]. Giraud et al., by addressing substitutions at specific positions of the indole ring system of these marine alkaloids, identified compound 55 (Table 3) as the most potent inhibitor of DYRK1A and CDC Like Kinase 1 (CLK1, IC_50_ = 0.034 µM and 0.032 µM, respectively), which are two PKs involved in alternative mRNA splicing and neurodegenerative pathologies [149].

**Table 3:** ATP-competitive DYRK1A inhibitors and their applications.

Chemical Structure	IC_50_ Values	Purpose/Biological Activities
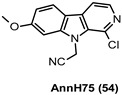	181 nM	Chemical probe for DYRK1A. Decrease of τ phosphorylation in cells [144].
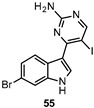	34 nM	Chemical tool [149].
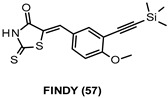	* IC_50_ data not available	DYRK1A inhibition at the translation level by suppressing autophosphorylation at Ser97 in a folding intermediate in cultured cells [150].
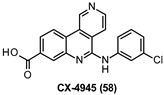	6.8 nM	Reversal of aberrant τ phosphorylation (Thr212) in DYRK1A-overexpressing mice. Inhibition of DYRK-1A-mediated phosphorylation of APP (IC_50_ ≈ 80 nM) and PS1 (IC_50_ ≈ 100 nM). Restoration of the neurological and phenotypic defects in an AD-like Drosophila model [151].
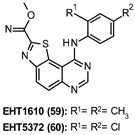	59: 0.36 nM 60: 0.22 nM	Chemical tools for DYRK1A-mediated diseases. Inhibition of DYRK1A-induced τ phosphorylation at multiple AD-relevant sites (Ser396, Thr212, Thr231). Normalization of Aβ-induced τ phosphorylation in neuronal cells. Normalization of DYRK1A-induced Aβ production in APP overexpressing cells [152,153].
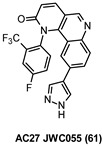	532 nM	Chemical tool useful for the design of optimized and novel agents to block τ phosphorylation [154].
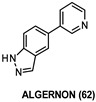	76.9 nM	Restoration of the proliferative capacity of NSCs derived from DS model mice. Suppression of τ phosphorylation in DYRK1A-overexpressing cells. Suppression of phosphorylation of endogenous τ in primary hippocampal neurons [57].

Ogawa et al. discovered 53 (Figure 11), a novel ATP-competitive inhibitor of DYRK1A bearing a benzothiazole moiety with IC_50_ and K_i_ values of 0.24 µM and of 0.18 µM, respectively. It was substantially free of MAOA inhibitory activity but showed submicromolar inhibitory activity against DYRK1B (IC_50_ = 0.23 μM) and, in an in vitro assay screening using a panel of 66 PKs, displayed >90% inhibition at 10 μM concentration on DYRK2, DYRK3, CLK1, CLK4, casein kinase 1 (CSNK1D), and PIM1. Notably, 53 and its acetylated derivative proINDY (56) effectively reversed the aberrant τ-phosphorylation at Thr212 in COS7 cells (a dose-dependent effect was observed for 53 in the 0.3–30 µM concentrations range), suggesting the useful role of these chemical tools in investigating the DYRK1A implication in τ-aggregate formation [51].

FINDY (57, Table 3) is a small molecule selected by Kii et al. among compounds of an internal chemical library by using a cell-based assay, named SPHINKS (substrate phosphorylation by sequential induction of kinase and substrate), to identify compounds able to selectively inhibit transitional intermediates of DYRK1A. In the folding process of this PK, an intermediate autophosphorylates Tyr319/321 and subsequently Ser97 in an intramolecular manner, which prevents DYRK1A degradation. Autophosphorylated DYRK1A takes on a mature conformation. Notably, 57 at 10 µM proved to suppress Ser97 autophosphorylation of a folding intermediate by preventing the incorporation of ATP, leading to DYRK1A degradation and decreasing the endogenous DYRK1A amount in a primary culture of cortical neurons. However, this compound did not inhibit substrate phosphorylation catalyzed by the mature kinase. Remarkable, 57 at 10 µM showed good DYRK1A selectivity over a panel of 275 kinases, since only five additional kinases (GSK-3β, MARK4, PIM1, PIM3, PLK3) were inhibited by over 75%, and none of these showed over 85% inhibition. Furthermore, no inhibitory effect was observed on DYRK1B and DYRK2 in an in vitro kinase assay [150].

CX-4945 (58, Table 3) is an ATP-competitive DYRK1A inhibitor (IC_50_ = 6.8 nM) with an in vitro potency much higher (about 20-fold) than that of 4, 53, or 56. Compound 58, due to its inhibitory activity against CDC2like kinases (CLKs), has been also involved in phase I and II clinical trials for cancer treatment and showed a safe profile. However, this agent proved to potently inhibit additional DYRK-family proteins (IC_50_ = 6.4, 18, and 1500 nM for DYRK1B, DYRK3, and DYRK4, respectively). Remarkably, 58 at 75 mg/Kg effectively reversed the aberrant phosphorylation of τ at Thr212 in DYRK1A-overexpressing mice and inhibited DYRK1A-mediated APP and PS1 phosphorylation in 293T cells with estimated IC_50_ values of ≈80 and 100 nM for APP and PS1, respectively. Moreover, 58 significantly restored the neurological and phenotypic defects in an AD-like *Drosophila* model, demonstrating the great potential of this inhibitor as a disease-modifying agent for AD [151].

EHT1610 and EHT5372 (59 and 60, respectively, Table 3), two methyl 9-anilinothiazolo[5,4-*f*]quinazoline-2-carbimidates derivatives identified by Chaikuad et al., present subnanomolar and selective DYRK1A/B inhibitory activity with a noncanonical binding mode at the ATP pocket of both enzymes [152]. Derivative 60 with an IC_50_ of 0.22 nM proved to be more potent than 4, 51, and 52 and displayed a high degree of selectivity over 339 kinases. The same compound inhibited DYRK1A-induced τ phosphorylation at multiple AD-relevant sites, including Ser396, Thr212, and Thr231 in biochemical assays, cell lines (e.g., HEK293 cells), and primary cortical neurons without affecting cell viability. Moreover, it normalized Aβ-induced τ phosphorylation in neuronal cells at 5 and 10 µM concentrations and DYRK1A-induced Aβ production in APP over-expressing cells (IC_50_ = 1.06 µM) emerging as a promising τ and amyloid-directed agent to alter the onset or progression of AD and other tauopathies [153].

The substituted 1,6-phenanthroline core of AC27 (JWC-055, 61, Table 3) was identified by Czarna et al., as an interesting chemical scaffold to support the design of optimized and novel DYRK1A inhibitors to block τ phosphorylation. It was selected among twenty-two different compounds, in turn selected among 1000 compounds of an internal library, and tested for their ability to inhibit and bind to DYRK1A. Compound 61 was found to strongly inhibit the phosphorylation of DYRKtide by DYRK1A with K_i_ and IC_50_ values of 252 nM and 532 nM, respectively. According to X-ray data, the same compound did not establish hydrogen bonds with the hinge of the enzyme, since bridging water between the hinge and the inhibitor was observed. In the pT212-τ phosphorylation assay, 61 proved to inhibit DYRK1A in NCI-H1299 cells, however, with a potency lower than in vitro activity. Finally, 100 nM concentration of 61 proved to be nearly 50 times more active than 4 in a NFAT Luc reporter assay [154].

Similar to 61, and additional PKs inhibitors reported in this review, ALGERNON (altered generation of neurons, 62, Table 3) emerged as the most promising derivative within a focused library of compounds promoting the growth of neural stem cells (NSCs) through DYRK1A inhibition. This compound, able to restore the proliferative capacity of NSCs derived from DS model mice, proved to inhibit DYRK1A in the mid-nanomolar range (IC_50_ = 76.9 nM) without the promiscuity of 51 or side effects of 4 due to low inhibition of MAO-A (IC_50_ = 2273.91 µM). Remarkably, 62 in the range of concentrations of 0.2–5 µM suppressed τ phosphorylation in DYRK1A-overexpressing cells in a dose-dependent manner and the phosphorylation of endogenous τ in primary hippocampal neurons, confirming its therapeutic potential for a wide range of disorders involving progressive or permanent neuronal loss such as neurodegenerative diseases and traumatic brain injury [57].

## 12. Multi-Target Compounds with Potential CNS Application

Almost twenty years ago, a few pioneering groups reported the possibility of efficiently exploiting the multi-target profiles of small organic molecules to tackle several complex and incurable pathologies as CNS-related diseases [155]. In this context, polypharmacology-based strategies and primarily multi-target-directed ligands (MTDLs) have triggered the interest of drug discovery community, both in academia and pharmaceutical companies, offering new paradigms with the potential to overcome some of the major limitations of classic “one target, one drug” strategies [156,157]. Several GSK-3β and DYRK1A-directed MTDLs have been developed as disease-modifying agents to combat different neurodegenerative and neuromuscular disorders. 

In the context of AD, Holzer et al. developed benzofuropyridine-based triple inhibitors of GSK-3β, CDK1, and CDK5, which are three prominent kinases related to τ pathology [58]. Among these PKs, CDK-5 has been found to be abnormally activated in AD. In contrast to the majority of cyclin-dependent kinases (CDKs), which promote cell cycle progression in proliferating cells, CDK5 is activated in post-mitotic neurons via the neuron-specific activator p35 to form the complex CDK5-p35, which plays a critical role in brain development and physiological synaptic activity.

**Figure 12:**
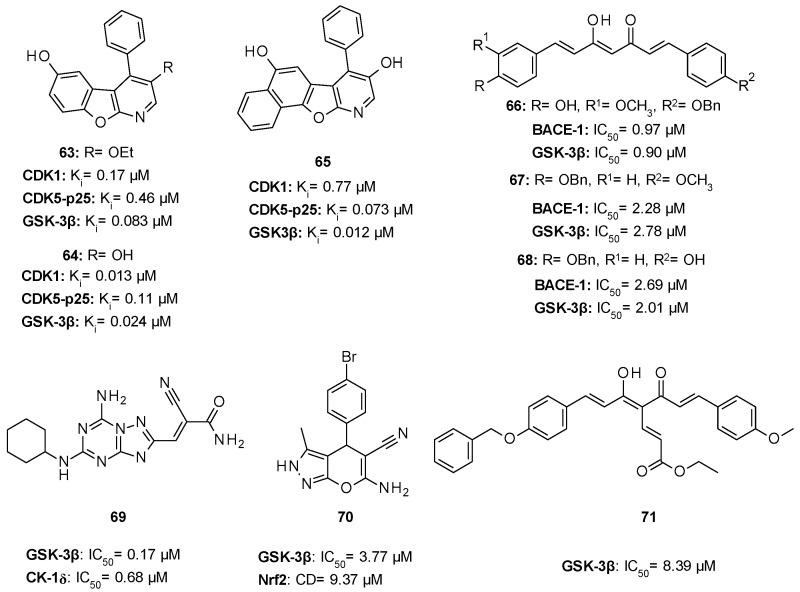
Chemical structures of multi-target GSK-3β inhibitors.

In AD brains, p25, the *N*-terminal truncated form of p35 generated by cleavage with calpain, is responsible for CDK5 overactivation, thus contributing to τ hyperphosphorylation [59]. Moreover, recent lines of evidence have demonstrated the crucial role of hyperactivated CDK5 in promoting aberrant CDK1 activation, which in turn induces neuronal death and potentiates the AD pathology. Among all synthetized triple GSK-3β/CDK1/CDK5 ATP-competitive inhibitors, the best results were obtained with analogs 63–65 (Figure 12), which showed submicromolar or nanomolar affinity toward two kinases and nanomolar or submicromolar affinity toward the third selected target. In detail, the 3-ethoxy derivative 63 displayed submicromolar affinities for CDK1 and CDK5 (K_i_ = 0.17 µM for CDK1 and 0.46 µM for CDK5) and nanomolar affinity for GSK-3β (K_i_ = 0.083 µM); the 3-hydroxy analog 64 emerged as the most interesting triple inhibitor, reaching affinities in the nanomolar range for CDK1 and GSK-3β (K_i_ = 0.013 and 0.024 µM, respectively) and in the submicromolar one for CDK5 (K_i_ = 0.11 µM); the best nanomolar affinities toward CDK5 and GSK-3β were obtained with compound 65 (K_i_ = 0.073 and 0.012 µM, respectively), which also showed submicromolar affinity for CDK1 (K_i_ = 0.77 µM).

Among them, 64 showed selectivity versus several PKs, since an absence of activity was observed against members of the PKA family (PKC-α, -γ, -ε, -iota), the receptor tyr kinase family (VEGFR2, ErBB2 and TIE2), WEE1, and CK1α; meanwhile, high micromolar values of activities ranging from 230 to 829 µM were shown against CDK6, EGFR of the receptor tyr kinase, and Src of the SRC family. Notably, derivative 63 proved to reduce τ phosphorylation by 61% at 8 µM concentration in a τ protein phosphorylation assay, in which AT180 monoclonal antibody was used to detect phosphorylated Thr231 and Ser235 sites in τ transfected COS-7 cells. In a split-luciferase assay, developed in house to study the effect of the novel triple inhibitors on the τ self-interaction in a human liver cell line (HuH-7), 64 and 65 showed a significant reduction of luminescence and therefore of τ interaction (38% and 29% inhibition at 1 µM, respectively; 71% and 84% inhibition at 10 µM, respectively). Derivative 63 caused only a mild inhibition of τ interaction at 1 µM (22%) and a higher inhibition up 65% at 10 µM [58].

Di Martino et al. developed dual BACE-1/GSK-3β inhibitors based on the curcumin scaffold having recognized both enzymes as two validated AD targets, whose concurrent inhibition could offer promise to achieve effective treatments. Among all compounds, derivatives 66–68 (Figure 12) emerged as well-balanced dual-target inhibitors with potency in the low micromolar range and proved to be brain permeable in the PAMPA-BBB assay, showing predictive penetration values in the CNS greater than or equal to 7 × 10^−6^ cm s^−1^. Among these derivatives, 66 emerged as a promising AD-modifying drug candidate to further develop due to its antioxidant effect by induction of NAD(P)H: quinone oxidoreductase 1 (NQO1 enzyme), which was accompanied by the absence of evident neurotoxic effects up to 20 µM and favorable pharmacokinetic behavior [61]. 

Redenti et al. developed triazolotriazine-based dual GSK-3β/CK-1δ ligands as potential neuroprotective agents useful to treat PD, taking into account the involvement of GSK-3β in microglial-mediated inflammation and of the delta isoform of CK1 family of Ser/Thr kinases (CK-1δ) in the neuroinflammatory process, mainly through the Wnt and Hedgehog pathways, along with the crucial role of both PKs in the hyperphosphorylation of τ, α-syn, and parkin. Among all derivatives, 2-cyanoacrylamide compound 69 (Figure 12) showed a submicromolar inhibitory activity against both selected targets (IC_50_ (GSK-3β) = 0.17 µM; IC_50_ (CK-1δ) = 0.68 µM). While a classical ATP competition was observed against CK-1δ, a covalent interaction between the cyanoacrylamide warhead of 69 and Cys199 of GSK-3β was confirmed by X-ray. In a PAMPA/BBB test, the same compound, due to the highly polar moieties, showed a permeability close to the limit of passively BBB-permeating compounds (Pe = 1.34 × 10^−6^ cm s^−1^). Remarkably, 69 did not display cytotoxicity up to 10 µM and prevented neurotoxin-induced cell death in a concentration-dependent manner in an in vitro models of PD (rat PC12 pheochromocytoma cells in the presence of neurotoxins 4-phenyl-1-methyl-1,2,3,6-tetrahydropyridine (MPTP) or 6-hydroxydopamine (6-OHDA)). In additional in vitro studies, in line with compelling evidence for a linkage between Wnt/β-catenin signaling and inflammatory events during PD progression, as well as GSK-3β upregulation and β-catenin degradation, compound 69 prevented 6-OHDA-induced cell death by inhibiting GSK-3β, and promoted β-catenin stabilization, thus restoring its neuroprotective potential [158].

Gameiro et al. designed and synthetized a new family of 2,4-dihydropyrano[2,3-*c*]pyrazoles as the first dual GSK-3β inhibitors/Nrf2 inducers considering the inverse correlation between the aberrant activation of GSK-3β and the decrease of antioxidant gene expression and cell defense effects due to Nrf2 downregulation. Among all derivatives, 70 (Figure 12) proved to be the most potent ATP-competitive GSK-3β inhibitor (IC_50_ = 3.77 µM) and one of the most potent Nrf-2 inducers. In a Nrf2-dependent luciferase reporter assay, 70 was able to increase luciferase activity in AREc32 cells with a CD (concentration required to double the basal luciferase reporter activity) value of 9.37 µM. Interestingly, cellular experiments in which GSK-3β was inhibited by a lithium salt (10 mM) and was silenced using siRNA demonstrated the independence of Nrf2 induction properties of compound 70 from its GSK-3β inhibitory activity. Moreover, fluorescence polarization and differential scanning fluorimetry assays indicated that the same compound was not able to inhibit the Nrf2-Keap1 interaction. Remarkably, in SH-SY5Y cells treated with okadaic acid (OA, 20 nM) to induce τ-hyperphosphorylation and aggregation through the selective inhibition of protein phosphatases PP1 and PP2A and cell death for oxidative stress increase (an in vitro AD model), 70 showed at 1 µM concentration a neuroprotection percentage over 50% [75]. 

Di Martino et al. by applying a hybridization strategy consisting of the introduction of a diethyl fumarate (DEF) fragment at the 4-position of the curcumin scaffold discovered compound 71 (Figure 12) as a brain permeable (Pe = 4.8 × 10^−6^ cm s^−1^ in a PAMPA-BBB Assay) dualistic GSK-3β inhibitor/Nrf2 inducer for PD treatment. In the Kinase-Glo assay, 71 showed low micromolar inhibitory activity against GSK-3β (IC_50_ = 8.39 µM), and kinetic studies suggested a non-ATP competitive mechanism of action. Moreover, the incubation of SH-SY5Y cells with curcumim-DEF hybrid 71 (5 µM) for 1, 3, and 6 h produced an increase of p-GSK-3α/β (Ser21/9) levels, suggesting an inhibition of GSK-3α/β activation. Concerning Nrf2 induction, the same hybrid derivative at 5 µM proved to induce the nuclear translocation of Nrf2 both after short-term (1 and 3 h) and long-term treatments (6 h) and showed superior effects to DMF, a well-known Nrf2 inducer, in augmenting Nrf2/ARE binding activity. Furthermore, in SH-SY5Y cells, 5 µM concentration of 71 significantly increased the mRNA levels of NQO1, a Nrf2 target gene, after 12 and 24 h of treatment. The same hybrid compound both in vitro and in vivo models of PD recorded very encouraging neuroprotective effects. In detail, pretreatment of SH-SY5Y cells with 71 (5 µM) mitigated the 6-OHDA-induced decrease in cell viability and significantly decreased the levels of toxic α-syn aggregates elicited by 6-OHDA in TagGFP2-α-syn SH-SY5Y cells. Furthermore, in a transgenic C. elegans model of PD, cotreatment with hybrid derivative 71 (5 µM) provided a partial rescue of the toxic effects induced by 6-OHDA with a decreased degeneration percentage (55%) of chefalic (CEP) neurons [36]. 

Similarly, to GSK-3β, the multi-target approach has been recently exploited to rationally design and synthetize multifunctional DYRK1A inhibitors. 

**Figure 13:**
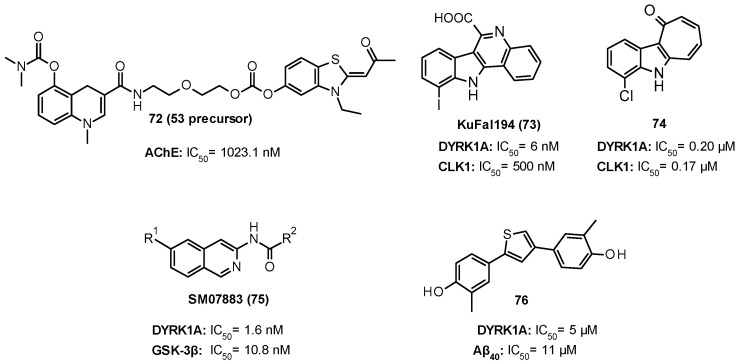
Chemical structures of multi-target DYRK1A inhibitors.

Barré et al. designed and synthetized dihydroquinoline 72 (Figure 13) as a promising bio-oxidizable prodrug to delivery both cholinesterase (ChE) and DYRK1A inhibitors for AD treatment [159]. Acetylcholinesterase (AChE) is a key enzyme in the CNS responsible for the hydrolytic metabolism of the neurotransmitter acetylcholine (ACh) into choline and acetic acid. It has proven to be a validate therapeutic target for symptomatic improvement in AD, since cholinergic deficit is a consistent and early finding in AD [160]. Accordingly, several inhibitors such as tacrine, donepezil, rivastigmine, and galantamine have been approved as symptomatic anti-AD agents. In this context, hybrid molecule 72 was devised by connecting through a carbonate linker the potent DYRK1A inhibitor 53 with a brain-penetrant bio-oxidative prodrug of a potent pseudo-irreversible AChE carbamate-based inhibitor, bearing an amino-PEG chain. This dihydroquinoline-based compound having demonstrated the ability to diffuse moderately in the CNS in the PAMPA BBB assay (Pe = 2.41 × 10^−6^ cm s^−1^) should be able to reach the brain, generating the corresponding quinonilium salt after bio-oxidation and 53 after carbonate linker hydrolysis. As expected, the bioprecursor 72 showed only a modest inhibitory activity against hAChE with micromolar IC_50_ value (1023.1 nM). On the contrary, the oxidized form displayed nanomolar potency on the same enzyme (IC_50_ = 81.4 nM). Moreover, the same compound did not display inhibitory activity against DYRK1A at 1 µM (10 µM ATP concentration) compared to 53 [159].

Lechner et al. starting from KuFal194 (73, Figure 13), a potent DYRK1A inhibitor (IC_50_ = 6 nM) with reasonable selectivity versus DYRK1B (IC_50_ = 600 nM) and CLK1 (IC_50_ = 500 nM), undertook an optimization campaign to identify improved DYRK1A inhibitors. Among all derivatives, particular attention was focused on [*b*]-annulated indole 74 (Figure 13), which emerged as well-balanced dual CLK1/DYRK1A submicromolar inhibitor (IC_50_ (CLK1) = 0.17 µM; IC_50_ (DYRK1A) = 0.20 µM) [161].

Melchior et al. identified SM07883 (75, Figure 13) as an interesting brain-penetrant dual DYRK1A/GSK-3β inhibitor (IC_50_ = 1.6 nM for DYRK1A and 10.8 nM for GSK-3β) in a kinase panel screen. Notably, this multi-target compound showed a reduction of phosphorylation of multiple τ epitopes, especially Thr12 site (EC_50_ = 16 nM) in cell-based assays, and in an anesthesia-induced transient τ hyperphosphorylation mouse hypothermia model, it showed reduced τ phosphorylation by 47% with the lowest dose of 1.25 mg/kg. Moreover, compared to the vehicle, a significant reduction of τ phosphorylation and aggregation was observed with alternative dose regimen of 75 leading to significantly lower numbers of τ-positive inclusions in brain stem and spinal cord samples [162]. The safety and tolerability of increasing doses of 75 have been also evaluated in a phase I clinical trial (ACTRN12619000327189) in healthy volunteers [163]. 

Ultimately, Mariano et al. reported the application of a focused multi-target approach to develop a novel class of selective dual inhibitors of DYRK1A and Aβ aggregation based on the bis(hydroxphenyl)thiophene scaffold. Among all derivatives, compound 76 (Figure 13) exhibited the best biological profile with a well-balanced inhibitory potency toward the selected targets (IC_50_ = 5 µM for DYRK1A) with Aβ_40_ inhibition (Aβ_40_ % inhibition at 100 µM = 91%; IC_50_ = 11 µM in a cell-free assay). The same compound proved to inhibit DYRK1A-catalyzed τ phosphorylation in stably transfected HEK293 cells and due to its favorable physicochemical properties might be potentially applicable to in vivo AD models [164]. 

## 13. Conclusions

Developing drugs for CNS remains the most challenging area in drug discovery, which is accompanied with the long timelines and high attrition rates. Nowadays, there is an ever-growing need to find drugs able to reach the brain in an adequate concentration for engaging CNS targets and modulating complex and interconnected signaling pathways linked to different neurological disorders. There is a plethora of CNS-related diseases with more complex pathological mechanisms, for which rationally designed multi-target compounds could offer higher efficacy and safety compared with single-target small-molecule ligands and overcome the same limits of mono-target drugs. In this scenario, GSK-3β, FYN, and DYRK1A are three attractive closely related PKs widely investigated within the neurokinome context due to their involvement in the pathophysiology of both neurodegenerative and neuromuscular disorders. Herein, we reported an overview of the most common neuronal pathological events and the crucial roles of all three PKs in different neurodegeneration pathways. We also included a deep analysis of the last decade from 2010 to 2020 literature focused on the development of novel mono- and multi-target GSK-3β, FYN, and DYRK1A inhibitors to overcome some limitations of known inhibitors and discover improved brain permeable modulators with drug-like properties. 

## Abbreviations

AChE	acetylcholinesterase
AD	Alzheimer’s disease
ALS	amyotrophic lateral sclerosis
APP	amyloid precursor protein
ARE	antioxidant response element
ASF	alternative splicing factor
Aβ	amyloid-β
BACE-1	β-site APP cleaving enzyme
BBB	blood–brain barrier
CDKs	cyclin-dependent kinases
CEP	chefalic
CLKs	CDC2-like kinases
ClogD	calculated distribution coefficient at pH 7.4
ClogP	calculated partition coefficient
CMCTs	canine mast cell tumors
CNS	central nervous system
DM1	myotonic dystrophy type 1
DSCR	Down syndrome critical region
DYRK1A	dual-specificity tyrosine phosphorylation-regulated kinase 1A
FDA	Food and Drug Administration
FTLD	frontotemporal lobar degeneration
GSK-3	glycogen synthase kinase-3
HD	Huntington’s disease
HMK	halomethylketone
ITDZs	5-imino-1,2,4-thiadiazoles
Keap1	Kelch-like ECH-associated protein 1
LTD	long-term depression
LTP	long-term potentiation
MAPKs	mitogen-activated protein kinases
MND	motor neuron disease
MTDLs	multi-target-directed ligands
*M*_W_	molecular weight
NF-kB	nuclear factor-kB
NFTs	neurofibrillary tangles
NMDARs	*N*-methyl-*D*-Aspartate receptors
PAMPA	parallel artificial membrane permeability assay
PD	Parkinson’s disease
P-gp	P-glycoprotein
PiD	Pick’s Disease
Pk_a_	acid dissociation constant
PKs	protein kinases
PP1	protein phosphatase 1
PrP^c^	cellular prion protein
PS1	presenilin 1
PSD	postsynaptic density
PSP	progressive supranuclear palsy
PTMs	post-translational modifications
SCIs	substrate competitive inhibitors
SFKs	Src family kinases
SH	Src homology
SMA	spinal muscular atrophy
SMN	protein survival of motor neuron
TDZDs	thiadiazolidindiones
TK	tyrosine kinase
α-syn	α-synuclein
